# Rational Mutagenesis of Cyclodextrin Glucanotransferase at the Calcium Binding Regions for Enhancement of Thermostability

**DOI:** 10.3390/ijms13055307

**Published:** 2012-04-25

**Authors:** Poh Hong Goh, Rosli Md. Illias, Kian Mau Goh

**Affiliations:** Faculty of Biosciences and Bioengineering, Universiti Teknologi Malaysia, 81310 Skudai, Johor, Malaysia; E-Mails: gph_ashin@hotmail.com (P.H.G.); r-rosli@utm.my (R.M.I.)

**Keywords:** CGTase, thermostable enzyme, site-directed mutagenesis, protein engineering, calcium binding site

## Abstract

Studies related to the engineering of calcium binding sites of CGTase are limited. The calcium binding regions that are known for thermostability function were subjected to site-directed mutagenesis in this study. The starting gene-protein is a variant of CGTase *Bacillus* sp. G1, reported earlier and denoted as “parent CGTase” herein. Four CGTase variants (S182G, S182E, N132R and N28R) were constructed. The two variants with a mutation at residue 182, located adjacent to the Ca-I site and the active site cleft, possessed an enhanced thermostability characteristic. The activity half-life of variant S182G at 60 °C was increased to 94 min, while the parent CGTase was only 22 min. This improvement may be attributed to the formation of a shorter α-helix and the alleviation of unfavorable steric strains by glycine at the corresponding region. For the variant S182E, an extra ionic interaction at the A/B domain interface increased the half-life to 31 min, yet it reduced CGTase activity. The introduction of an ionic interaction at the Ca-I site via the mutation N132R disrupted CGTase catalytic activity. Conversely, the variant N28R, which has an additional ionic interaction at the Ca-II site, displayed increased cyclization activity. However, thermostability was not affected.

## 1. Introduction

The industrial enzyme cyclodextrin glucanotransferase (CGTase, EC 2.4.1.19) catalyzes hydrolysis, disproportionation, coupling and cyclization reactions [1]. Cyclization is the major activity of CGTase, where starch is converted into cyclodextrins (CDs). Cyclodextrins offer broad biotechnological applications. A recent review summarizes these applications and work related to previous protein mutagenesis studies [1]. In 1992, the first study regarding the manipulation of CGTase product specificity was reported [2]. The authors suggested that hydrophobic aromatic residues, such as Phe191 and Phe255, could affect product specificity. Following that study, attempts to elucidate the importance of the substrate binding subsites, particularly subsites −3, −6, and −7, were also reported [3,4]. A mutation at position 179 in subsite −6 in *Bacillus circulans* DF 9R CGTase both altered the CD ratio and affected catalytic efficiency [5]. By substituting the histidine with a smaller amino acid at position 43 (subsite −3) in *Bacillus* sp. G1, the γ-CD yield was enhanced [6]. Ample mutagenesis reports have discussed the theme of product specificity.

Thermostable CGTase is particularly important due to the requirement of high operating temperatures in starch processing. Solutions to overcome this temperature limitation have been sought. First, numerous outstanding thermostable CGTases have been cloned from thermophiles. Most likely, the stability of CGTase is contributed by ionic interactions [4]; unfortunately, the exact location that can improve the thermostability has yet to be determined. Second, CaCl_2_ is added to CGTase to promote activity and stability [7,8]. However, the removal of CaCl_2_ during downstream processing is tedious. Third, immobilizing CGTase on suitable carriers may also improve thermostability [9,10]. Fourth, CGTase thermostability can be improved using a protein engineering approach [11]. The stability of *Bacilus circulans* 251 CGTase was significantly increased by introducing a new salt bridge at the protein surface in domain B. To the best of our knowledge, this is the only study that mutated CGTase to improve thermostability. In fact, research on elucidating the protein structure and CGTase thermostability is limited. Recently, *in silico* mutations and molecular dynamic simulations were used to investigate the relationship between thermostability and salt bridges [12]. The computational simulations suggest that thermostability of *Bacillus macerans* CGTase might be improved by introducing new salt bridges into thermally unstable regions.

Aforementioned, addition of CaCl_2_ can improve CGTase stability. Studies related to the modification of calcium binding sites of CGTase are scarce. Unlike CGTase, numerous mutational efforts were performed at the calcium binding sites of α-amylase, which demonstrated that the highly conserved calcium-interaction residues influence thermostability [13–15]. α-amylase and CGTase belong to the α-amylase family 13 and they share several common characteristics: (i) possess a catalytic TIM-barrel fold domain, (ii) are able to act on α-glycosidic linkages, (iii) employ a similar catalytic mechanism that is α-retaining double displacement and (iv) possess Asp, Glu and Asp as catalytic residues [16]. Certain α-amylases possess three calcium binding sites and one sodium binding site [13], while most reported CGTases possess only two calcium binding sites located at domain A and the A/B domain interface [17]. Mutations at calcium binding sites caused detrimental effects to both α-amylase stability and catalytic activity [13,15]. For instance, in *Bacillus licheniformis* α-amylase (BLA), the mutation D204K at calcium binding site II caused a decrease in the T_50_ (temperature for 50% inactivation) from 83 °C to 64 °C, and the activity half-life at 85 °C decreased from 5 min to less than 1 min [13]. Substitution of a calcium-interaction residue in *Bacillus amyloliquefaciens* α-amylase (BAA) also resulted in a reduction in catalytic activity and thermostability [15]. In contrast, a mutagenesis study of *Bacillus stearothermophilus* US100 demonstrated that shortening the length of a loop adjacent to calcium binding site II increased the stability of the α-amylase [18]. In these amylases, the effects of mutagenesis on the calcium binding sites are distinctive in each example of interest. As limited information on the influence of CGTase mutagenesis on thermostability is available, the data presented herein may provide important information for future studies relating to the CGTase calcium binding regions.

## 2. Results and Discussion

### 2.1. Mutagenesis Design

For many years, our group has been working with a CGTase from *Bacillus* sp. G1 (GenBank Accession number: AY770576) [19]. The wild-type CGTase is an alkalotolerant protein and exhibits an optimum temperature of 60 °C. Earlier, several mutagenesis experiments were performed on CGTase G1 for the study of product specificity. Double mutations at positions H43T and Y87F had more outstanding properties than the other constructed mutants [6]. This work aims to further improve the thermostability of this mutant using a mutagenesis approach. All mutagenesis reported herein was performed on this mutant gene. For clarity, throughout this report, designation of “parent CGTase” refers to the starting CGTase source, *i.e.*, the mutant CGTase H43T/Y87F [6]. Table 1 summarizes the comparison of parent CGTase with some known CGTases.

Strategies to protein engineer CGTase for altered activity and product specificity are well reviewed [1], but only one work successfully used mutagenesis to increase temperature stability. In this study, we inferred that amino acid substitution at the two calcium binding regions would alter thermostability. The main motivation for this mutation is that no study has elucidated the importance of calcium binding sites to CGTase thermostability using a protein engineering approach.

**Figure 1:**
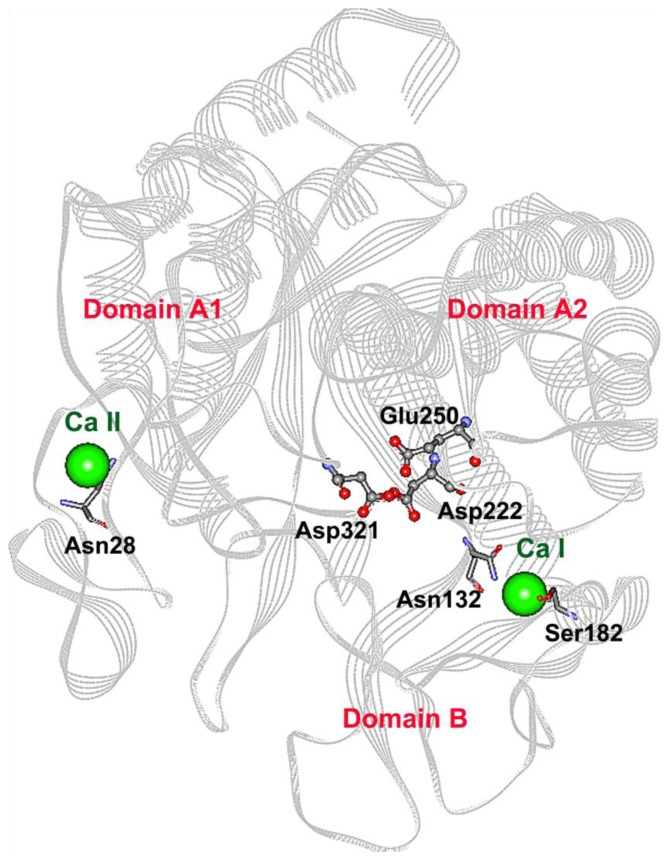
Theoretical 3D structure of the parent CGTase used in this work. For simplicity, Domain C, D and E are not shown. The catalytic residues Asp222, Glu250 and Asp321 are presented in scaled ball and stick. The two calcium ions are represented by green balls. The calcium binding sites I and II are located at the A/B domain interface and domain A, respectively. The residues for which point mutations were made (Asn28, Asn132 and Ser182) are displayed in stick.

Four CGTase variants (S182G, S182E, N132R and N28R) were constructed. The mutation site S182 was chosen based on a primary sequence comparison with a thermostable CGTase. Residue 182 is located in an amino acid stretch important for thermostability [11]. In contrast, the mutations N132R and N28R were located at calcium binding sites I and II, respectively. By changing Asn to the charged residue Arg, an ionic interaction with an oppositely charged residue would be introduced to the calcium binding pockets. This newly introduced interaction would hopefully stabilize the CGTase. The detailed reasons and the effect of these mutations on thermostability are discussed in the following sections. Figure 1 is a schematic diagram showing the location of each mutation sites.

### 2.2. Activity Screening and Purification of Parent and Mutant CGTases

**Figure 2:**
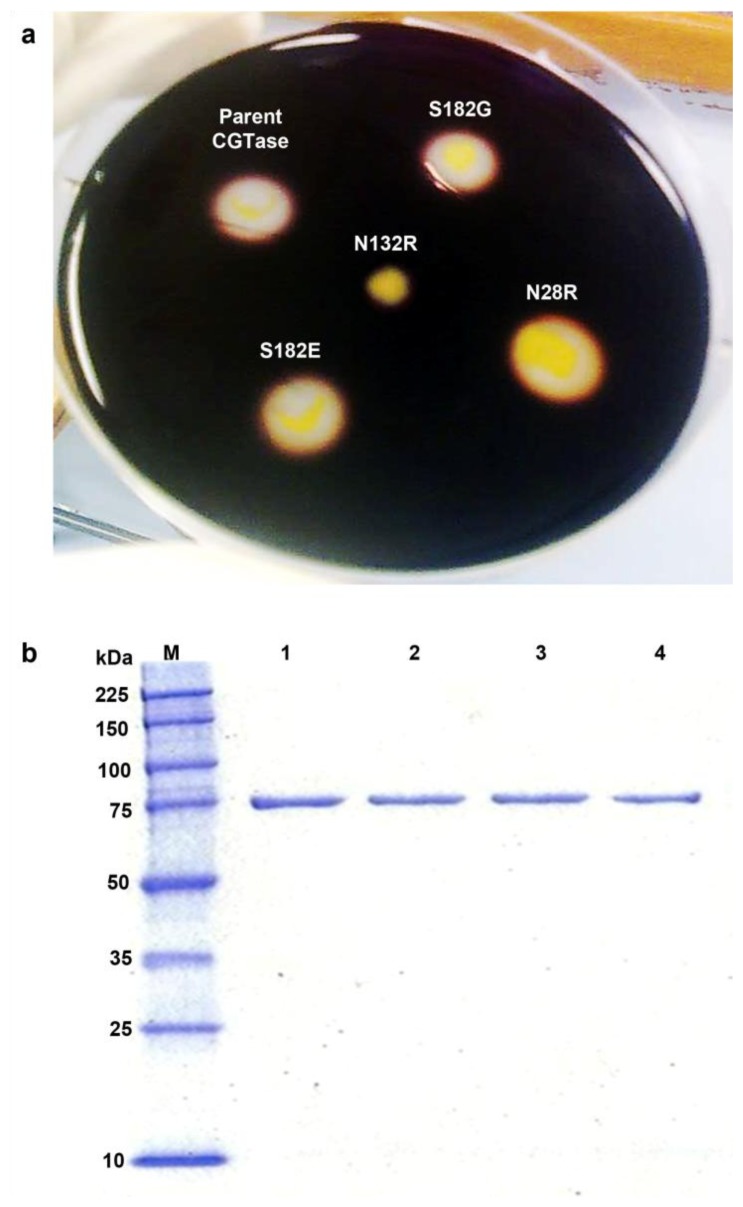
(**a**) Halo zone detection on starch agar plates. (**b**) SDS-PAGE. Lane M, molecular weight standards; Lane 1, purified parent CGTase; Lane 2, purified mutant N28R; Lane 3, purified mutant S182G; Lane 4, purified mutant S182E.

The parent CGTase gene and four constructed mutant genes were cloned into the pET-22(b+) system and expressed in *E. coli* BL21 under the control of the T7 promoter. From preliminary activity screening on a starch agar plate, the mutation N132R was detrimental, and activity was lost. The three other mutants retained starch-degrading activity similar to the parent CGTase, and they formed observable halo zones around colonies on starch plates flooded with a 1% iodine solution (Figure 2a). The screening was repeated five times and consistent results were obtained. Purification of the parent and mutant CGTases was performed, and highly pure enzyme was obtained with a molecular mass of approximately 75 kDa, as determined by SDS-PAGE (Figure 2b). This level of purity was achieved because of the native binding affinity between α-CDs and the binding sites in CGTase. The purified CGTases were then used for biochemical characterization.

### 2.3. Mutations Adjacent to Calcium Binding Pocket I, S182G and S182E, Affected CGTase Thermostability

**Figure 3:**
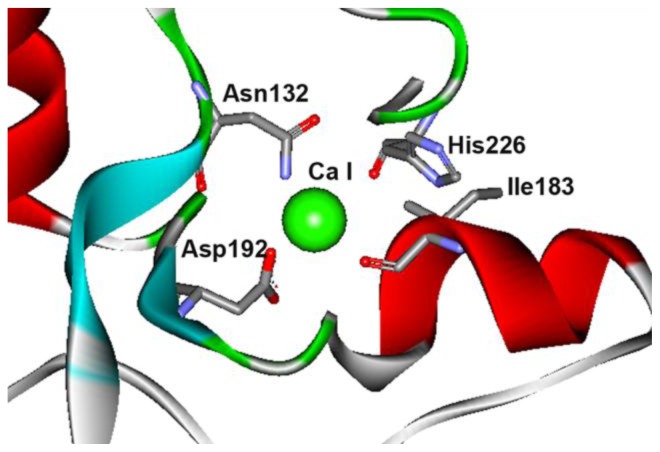
Computational model for calcium binding site I at A/B domain interface. Numbering is based on the parent CGTase used in this work.

**Figure 4:**
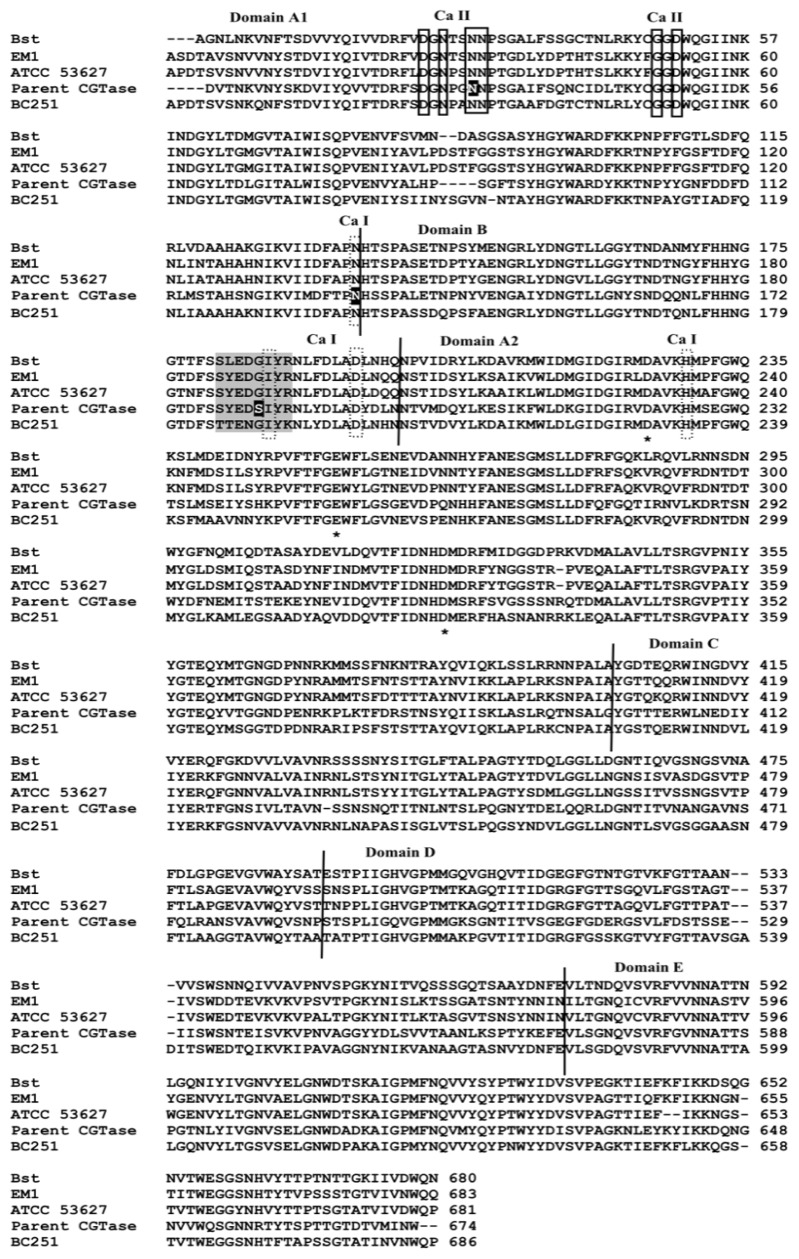
Amino acid sequence alignment between *B. stearothermophilus* CGTase (denoted Bst; PDB ID: 1CYG) *Thermoanaerobacterium thermosulfurigenes* EM1 CGTase (EM1; 1CIU), *Thermoanaerobacter* sp. ATCC 53627 CGTase (denoted ATCC 53627), *B. circulans* 251 CGTase (BC251; 1CXI) and parent CGTase (current work). The calcium interacting residues at sites I and II are bordered by a dash and solid rectangular boxes, respectively. The residues marked with asterisks are the important catalytic residues of CGTase. The shading portion refers to the region that has been shown to be important for thermostability [11]. The positions of point mutations in this study are highlighted.

Domain B of the enzyme is generally thought to contribute to the stability of CGTase and α-amylase [11,13]. Three domain B residues (Ile183, Asp192 and His226) and Asn132 from domain A are the residues that interact with calcium at site I (Figure 3). These residues are folded in close proximity at the A/B domain interface. Ile183 is located within a conserved segment of amino acids (178-SYEDSIYR-185) in parent CGTase. This region of sequence is conserved in the thermostable CGTase ATCC 53627 and EM1 (Figure 4). In the thermolabile CGTase BC251, the respective sequence is 185-TTENGIYK-192. Interestingly, this region has been shown to be important for thermostability in the BC251 CGTase. Altering the whole 185-TTENGIYK-192 segment in the CGTase BC251 to resemble the sequence 186-SYEDGIYR-193 in the thermostable EM1 CGTase increased the activity half-life to 73 min from 9.3 min for the native enzyme [11].

**Figure 5:**
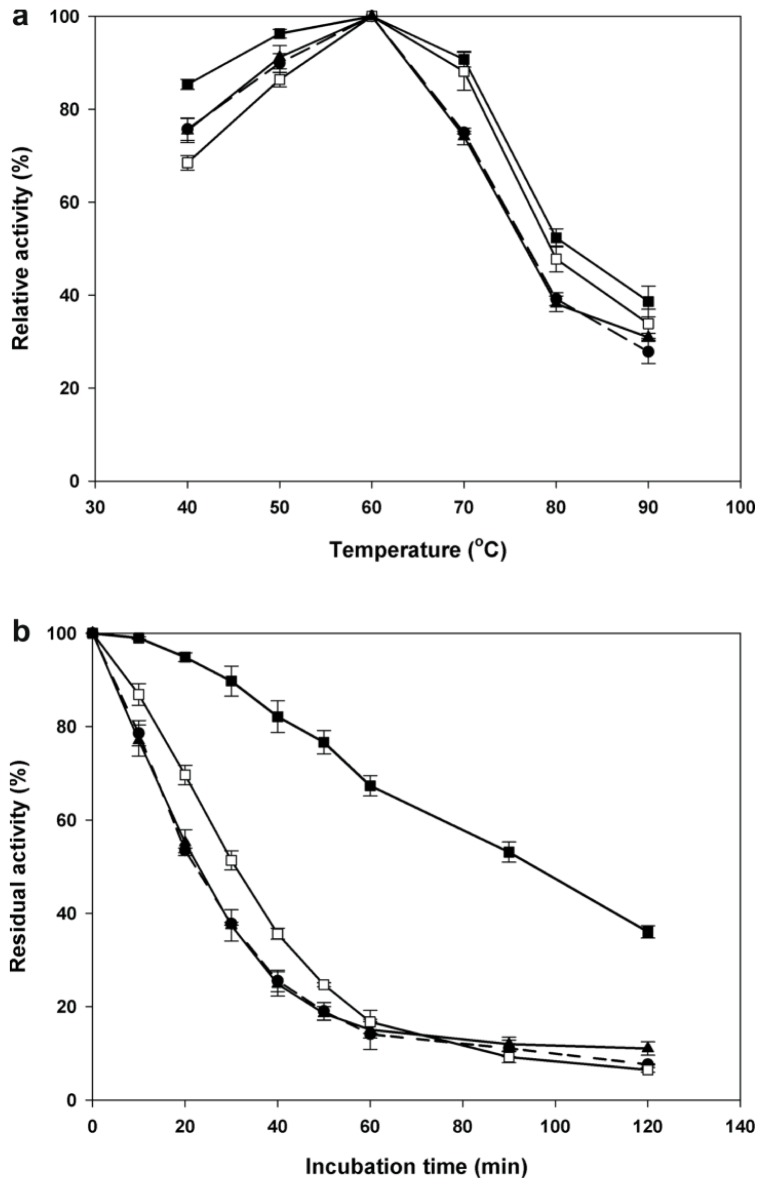
Effects of temperature on the activity and stability of the parent CGTase (●, dashed line), mutant N28R (▲, solid line), mutant S182G (■, solid line) and mutant S182E (□, solid line). (**a**) Optimal temperature. (**b**) Time plot for CGTase thermostability at 60 °C up to 120 min.

The parent CGTase has a distinctive residue at position 182, but the thermostable CGTases have a glycine residue in this position (Figure 4). Hence, the mutant S182G was constructed, and the effect of this mutation on enzyme stability and activity was examined. Similar to the parent CGTase, the S182G mutant is most active at 60 °C, yet it exhibited tolerance to a broader range of temperatures (Figure 5a). At 60 °C, the half-life (94 min) of the S182G mutant was approximately 4.3-fold higher than that of the parent CGTase (22 min) (Figure 5b). Substitution of the serine residue to glycine affected the catalytic performance. The β-cyclization specific activity, the major activity of CGTase, was decreased by 25% to 162 U mg^−1^ (Table 2), while the *k*_cat_ was also reduced from 3.13 s^−1^ to 2.62 s^−1^ (Table 3). Nevertheless, the catalytic efficiency (*k*_cat_/*K*_m_) of the S182G mutant (2 mL mg^−1^·s^−1^) was better than that of the native CGTase (1.82 mL mg^−1^·s^−1^).

Part of the conserved thermostability region (amino acids 179–184) folded as a short α-helix and is in close proximity to the active cleft at the A/B domain interface. *In silico* modeling suggests that the single replacement of serine with glycine shortened the α-helix length by reducing the number of α-helix forming residues from six to five. The CGTases from *Bacillus stearothermophilus* (PDB ID: 1CYG) and *Thermoanaerobacterium thermosulfurigenes* EM1 (1CIU) have five and four residues at the α-helix, respectively, whereas mesophilic CGTases from *Bacillus circulans* 251 (1CXI) and *Bacillus* sp. 1011 (1PAM) comprise six residues. Thus, a shorter α-helix at this region may be favorable for CGTase stability. An amino acid with a β-carbon may cause steric clashing between the β-carbon and the peptide backbone [23,24]. Among the amino acids, glycine has the simplest side chain and lacks a β-carbon and therefore exhibits a higher torsional freedom. The replacement of serine to glycine may possibly relieve unfavorable conformational strain and steric interactions in the folded α-helix. A similar substitution of serine to glycine increased the thermostability of *Clostridium thermocellum* endoglucanase, where the activity half-life was increased by eight-fold at 85 °C [23].

As residue 182 was recognized as a determining residue for CGTase stability, another mutation was made: Ser182 was replaced with a negatively charged Glu. The S182E mutant was more stable and active than parent CGTase in the temperature range of 70–90 °C (Figure 5a). Furthermore, the activity half-life at 60 °C was slightly enhanced to 31 min (Figure 5b), and this increment is statistically significant with a 95% confidence interval based on T-test analysis. This enhancement in stability might be caused by the additional cross-domain ionic interaction formed between Glu182 (domain B) and Lys225 (domain A2) that was predicted from the homology model. Lys225 is one of the substrate binding residues at subsite +2. The extra ionic interaction caused an interruption to CGTase catalysis and caused a decrease in the specific activities and *k*_cat_ values. The β- and γ-cyclization specific activities were reduced by 18% and 30%, respectively (Table 2). The *k*_cat_ value decreased approximately 1.2-folds to 2.57 s^−1^, whereas the catalytic efficiency retained similar to parent CGTase (see Table 3).

### 2.4. The N132R Mutation at the Calcium Binding Site I Caused Enzyme Dysfunction

**Figure 6:**
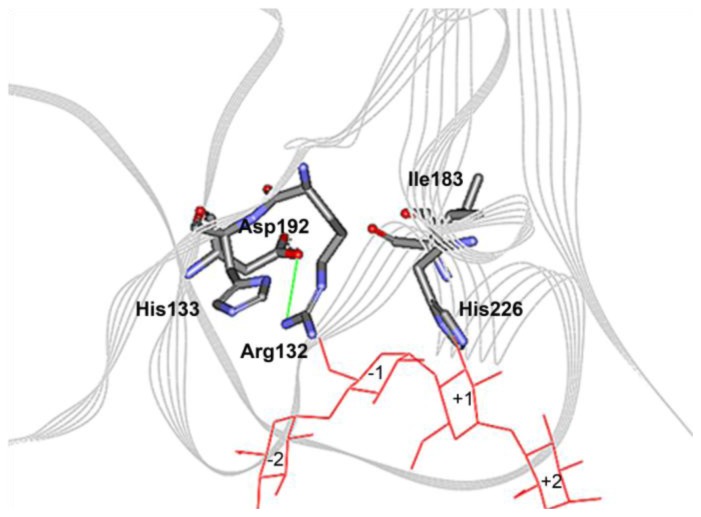
Theoretical model for calcium binding site I with mutation N132R. An ionic interaction is formed between Arg132 and Asp192, yet the long arginine side-chain could possibly block the interaction of His133 (subsite −1 residue) with the substrate (maltotetraose).

The aforementioned Ser182 and its mutant derivatives are adjacent to a calcium-interacting residue (Ile183) at the calcium binding site I. Asn132 and Asp192 are two other calcium interacting residues at Ca-I site (Figure 3). Asn132 was replaced with a positively charged Arg. We intended to introduce an ionic interaction between residues Asn132 and Asp192 that are located in a β-turn and a β-sheet, respectively. We thought the ionic interaction could stabilize CGTase by pulling the two opposite secondary-structures together. Unfortunately, substitution of Asn132 to an Arg led to enzyme dysfunction. No activity could be detected on starch plates or by the CGTase activity assay. The Asn132 residue is a strictly conserved residue in CGTase and may be an amino acid that is intolerant to replacement. It is hypothesized that the long side-chain of the arginine points towards the substrate-binding subsite −1 and may cause a steric hindrance to His133 (Figure 6). His133 is important for binding to the glucose moiety of starch, and earlier mutagenesis studies demonstrated that it is a sensitive residue [25]. Substitution of His133 to Asn reduced the activity of the *Bacillus* sp. 1011 CGTase by 75%. Furthermore, replacement to Arg nearly inactivated the enzyme activity such that the activity was only 2% of the wild-type CGTase. Hence, any modification, disturbance or hindrance to the interaction between residue 133 and the substrate could critically affect the enzyme activity.

### 2.5. The N28R Mutation at the Calcium Binding Site II Affected CGTase Activity

**Figure 7:**
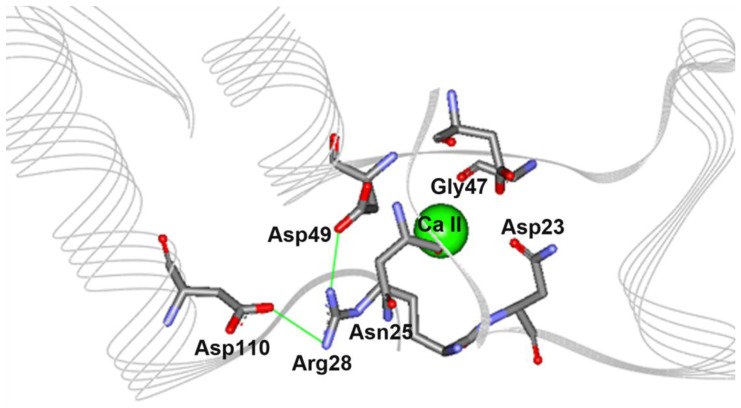
Theoretical structure for calcium binding site II with mutation N28R. An ionic triad Asp49-Arg28-Asp110 is formed due to the mutation.

The calcium binding site II of CGTase is located in domain A1 and contains six calcium interacting residues: Asp23, Asn25, Asn28, Asn29, Gly47 and Asp49. Calcium binding site II is found along a stretch of loops and short alpha helices that appear as a distorted U-shape where the calcium binds at the middle of the pocket (Figure 7). Similar to the approach used for the N132N mutation mentioned earlier, we intended to introduce ionic interactions into the pocket by mutating Asn28 to a positively charged Arg. Based on the protein model, the charged side chain of mutated Arg28 could form a triad salt-bridge with Asp110 and Asp49. The latter residue is a calcium interacting residue located on the opposite loop (Figure 7). The optimum temperature and activity half-life of this mutant were comparable with parent CGTase (Figure 5). Interestingly, the variant N28R exhibited a significantly increased enzyme activity. This mutant displayed approximately 1.7- and 2.0-fold increases in the β-CD and γ-CD cyclization activity, respectively (Table 2). This result is quite unexpected, as the loop containing the mutation does not directly participate in the catalytic reaction.

### 2.6. Product Analysis by HPLC

The action and preference of both the parent and mutant enzymes on five different types of starch (tapioca, corn, rice, potato and soluble starch) were investigated. The results from the HPLC analysis (Table 4) indicate that the efficiency of starch conversion to cyclodextrins is highly dependent on the substrate and are compatible to the findings of previous research [8,26]. All of the studied CGTases have a similar trend of substrate preferences. The researchers were able to produce the highest amount of cyclodextrins from the tapioca starch. This result may be because tapioca starch has lower amylose content and can be easily degraded by the starch-hydrolyzing enzymes. Overall, the S182G mutant has better performance in cyclodextrin production regardless of the type of starch used for the reaction. The S182G mutant has an improved stability and, therefore, leads to higher production. The analysis also suggests that all of the mutated sites are not important for altering the product specificity (data not shown).

## 3. Experimental Section

### 3.1. Bacterial Strains and Plasmids

The parent CGTase gene (H43T/Y87F CGTase gene) of this study is a mutant gene constructed from the *Bacillus* sp. G1 CGTase gene and was generated in an earlier study [6]. The complete parent and mutagenic CGTase DNAs constructed in this work were cloned into the pET-22(b+) system (Novagen, Darmstadt, Germany). *Escherichia coli* (*E. coli*) DH5α was used for cloning and selection of recombinant plasmids. The desired recombinant plasmid was then transformed into protease-deficient *E. coli* BL21 (DE3) (Novagen, Darmstadt, Germany) for enzyme production.

### 3.2. Site-Directed Mutagenesis

The CGTase mutants were generated by overlap extension PCR. All of the primers used in the PCR experiments, including the internal mutagenic primers, are described in Table 5. All PCR reactions were carried out using 0.3 μM of each primer, 0.2 mM of each dNTP, 1.5 mM MgSO_4_, and 1 U of KOD Hot Start DNA Polymerase (Novagen, Darmstadt, Germany) in 1× reaction buffer. The PCR experiments were performed using a Biorad MJ Mini™ gradient thermal cycler with 2 min of initial denaturation at 95 °C, followed by 30 cycles of denaturation at 95 °C, annealing at an appropriate temperature and extension at 70 °C. The desired PCR product was purified from a gel using the Wizard SV Gel and PCR Clean—Up System Kit (Promega, Madison, Wisconsin, USA). Clones carrying the mutant genes were identified using restriction enzyme analysis, and the respective mutations were verified by DNA sequencing (First Base Laboratories Sdn Bhd) using both pBRrevBam and T7 terminator primers and several internal oligonucleotides.

### 3.3. Production and Purification of Recombinant Enzymes

A single colony of *E. coli* BL21 (DE3) carrying the recombinant plasmid pET-22(b+) was grown in 50 mL of LB broth supplemented with 100 μg/mL of ampicillin (Calbiochem, San Diego, California, USA) at 37 °C overnight. The resulting cultures were centrifuged at 8200× g for 10 min, and the pellet was resuspended with fresh LB/amp broth. Next, the cell suspension was inoculated into 1000 mL of the same medium and cultured in a shaking incubator until the A_600nm_ = 1.0, at which point the production of the recombinant enzyme was induced by the addition of IPTG to a concentration of 0.1 mM. The cultures were incubated at 25 °C to reduce the formation of inclusion bodies.

The extracellular crude enzymes were obtained by centrifugation of cell cultures at 2000× g for 30 min and then precipitated with 70% ammonium sulfate. The resulting precipitate was dissolved in 10 mM sodium acetate buffer (pH 6.0) and then intensively dialysed against the same buffer to remove excess ammonium sulfate. The enzyme was further purified using an α-CD-bound, epoxy-activated Sepharose 6B affinity chromatography column with the protocols described by Sian *et al*. [8] with a slight modification. The affinity chromatography was conducted using an ÄKTAprime plus system with a flow rate of 0.5 mL/min throughout the purification process. A buffer (10 mM sodium acetate pH 5.5) containing 500 mM NaCl and 1% of α-CD (CycloLab Ltd, Budapest, Hungary) was used to elute the bound enzyme. The collected fractions were tested using a CGTase assay, and the active fractions were pooled and dialysed against a 100 mM phosphate buffer (pH 6.0).

### 3.4. SDS-PAGE

The molecular weight and purity of the purified enzymes were determined by sodium dodecyl sulfate-polyacrylamide gel electrophoresis (SDS-PAGE) using a 12% vertical slab polyacrylamide gel. Broad Range Protein Molecular Weight Markers (Promega, Madison, Wisconsin, USA) were used to estimate the molecular weight of the protein of interest. Samples were boiled at 100 °C for 5 min in 1× sample buffer containing 1% SDS and 1% β-mercaptoethanol. Electrophoresis was conducted at a constant current of 150 V for 1 h using 1× running buffer containing 0.1% SDS. After electrophoresis, the gel was stained with Imperial Protein Stain (Pierce Chemical, Rockford, Illinois, USA) for 2 h and then de-stained overnight with water.

### 3.5. Protein Determination

The protein concentration was quantified by using a BCA Protein Assay Kit (Pierce Chemical, Rockford, Illinois, USA) with bovine serum albumin as the standard. The measurement was carried out using the standard protocols provided by the manufacturer.

### 3.6. CGTase Assays

All CGTase assays were performed based on protocols described in earlier work [6]. The β- and γ-cyclization activities of CGTase were determined using a phenolphthalein assay and a bromocresol green (BCG) assay, respectively. The enzymatic activity for formation of β-CD was determined by incubating 0.1 mL of purified enzyme with 1.0 mL of 4% (w/v) soluble starch in 100 mM phosphate buffer (pH 6.0) at 60 °C for 10 min. The reaction was stopped by the addition of 3.5 mL of a 30 mM NaOH solution. Next, 0.5 mL of 0.02% (w/v) phenolphthalein in 5 mM Na_2_CO_3_ was added to the mixture and thoroughly mixed. The reaction mixture was left for 15 min at room temperature, and the reduction in color intensity was measured at 550 nm. One unit of enzyme activity was defined as the amount of enzyme that produced 1 μmol of β-CD per min under the assay conditions.

For the BCG assay, a reaction mixture containing 0.2 mL of the CGTase sample and 1 mL of 4% soluble starch in phosphate buffer pH 6.0 was incubated in a 60 °C water bath for 20 min. After the incubation, 0.5 mL of 200 mM HCl was added to stop the enzymatic reaction, and 0.2 mL of 0.05% (w/v) bromocresol green in 20% (v/v) ethanol was added and left at room temperature for 20 min. A volume of 2 mL of 1000 mM acetate buffer (pH 4.2) containing 30 mM citric acid was added to the reaction mixture prior to the measurement of color intensity at 630 nm. One unit of γ-CD forming activity was defined as the amount of enzyme that produced 1 μmol of γ-CD per min under the assay conditions.

In the coupling reaction, β-cyclodextrin (Cyclolab Ltd., Budapest, Hungary) and methyl-α-d-glucopyranoside (Sigma-Aldrich, St. Louis, Missouri, USA) were used as the donor and acceptor, respectively. A 0.1 mL volume of purified enzyme was added to a 1 mL solution containing 10 mM β-CD and 20 mM methyl-α-d-glucopyranoside. After 30 min of incubation at 60 °C, the coupling reaction was stopped by boiling for 10 min and cooling to room temperature. Next, 0.1 mL of the reaction mixture was treated with 4 U of glucoamylase (Sigma-Aldrich, St. Louis, Missouri, USA) at 50 °C for 1 h to convert the linear oligosaccharides produced from the coupling reaction into glucose units. The amount of glucose was accurately detected by the Glucose (GO) Assay Kit (Sigma-Aldrich, St. Louis, Missouri, USA) using the standard protocols suggested by the manufacturer. One unit of activity was defined as the amount of enzyme necessary to couple 1 μmol of β-CD per min under the assay conditions.

### 3.7. Effects of Temperature on Enzyme Activity and Stability

The optimum pH of mutant CGTases was determined by substituting the 100 mM phosphate buffer (pH 6.0) used in the standard CGTase assay with the following buffers: 100 mM sodium acetate buffer (pH 4.0–5.0), 100 mM sodium phosphate buffer (pH 6.0–8.0), 100 mM Tris-HCl (pH 9.0) and 100 mM glycine-NaOH buffer (pH 10.0). The pH stability was determined by incubating 0.1 mL of enzyme with 0.2 mL of the buffers mentioned above (pH 4.0–10.0) at room temperature for 30 min. The residual activity of the enzyme was then determined by the standard β-cyclization assay.

### 3.8. Kinetic Studies

The kinetic parameters were estimated by monitoring β-CD formation at 60 °C with various concentrations of soluble starch (0.5–8.0 mg·mL^−1^) as the substrate. The parameters of *K*_m_ and *V*_max_ were determined from a Michaelis-Menten plot using the SigmaPlot 11.0 program. The turnover number, *k*_cat_, of the enzyme of interest was determined by dividing the value of *V*_max_ with the concentration of enzyme (*k*_cat_= *V*_max_/[*E*]_0_).

### 3.9. HPLC Product Analysis

The production of cyclodextrins by CGTase using different starches (e.g., tapioca starch, soluble starch, rice starch, potato starch and corn starch) was determined by incubation of 0.1 mL of purified enzyme with 1% (w/v) starch in 20 mM sodium acetate buffer (pH 6.0) at 60 °C for 18 h. Maximum CD production happened at 18 h (data not shown). The reaction mixture was heated at 100 °C for 10 min to stop the enzymatic reaction. Insoluble particles were filtered out using a 0.45 μM syringe filter (Whatman, Clifton, New Jersey, USA). The amount of cyclodextrins produced by CGTase was determined by high-performance liquid chromatography (HPLC) with a Waters Spherisorb NH2 Column (5 μm, 4.6 × 250 mm). The mobile phase was 70% acetonitrile (HPLC grade, Merck, Darmstadt, Germany) with 30% nanopure water, and the flow rate of the mobile phase was maintained at 1 mL min^−1^. The column was kept at 30 °C, and a Waters 2414 refractive index detector (Waters, Milford, Massachusetts, USA) was used to detect the reaction products. High purity oligosaccharides, such as glucose, maltose, α-, β-, and γ-CD, were used as standards in this analysis.

### 3.10. Tertiary Structure Modeling

In this work, homology modeling and energy minimization were performed using the Accelrys Discovery Studio 2.5. The three-dimensional structures of the parent CGTase and the CGTase mutants were built with the crystal structure 1CYG (*Bacillus stearothermophilus* CGTase) as a reference structure. The structure 1CYG was selected because it has a good structure resolution and closest sequence identity to the parent CGTase of this work. The constructed models were minimized using an algorithm of steepest descent with harmonic constraints applied to restrict the polypeptide chain backbone. The quality of the minimized models was verified using the Structural Analysis and Verification Server (SAVES). The theoretical models were then superimposed with the crystal structure 1CXH (CGTase *Bacillus circulans* 251 complexed with ligands) [27] to show the location of these ligands in our models.

### 3.11. Protein Sequence Alignment

Primary sequence alignment between thermostable CGTases (*Thermoanaerobacterium thermosulfurigenes* EM1, *Thermoanaerobacter* sp. ATCC 53627 and *Bacillus stearothermophilus* CGTases) and mesophilic CGTases (*Bacillus circulans* 251 and parent CGTase in this study) was performed using the ClustalW2 program [28]. The calcium binding residues in parent CGTase were identified based on a previous study [17].

## 4. Conclusions

The two calcium binding sites are important for the stability and catalytic activities of CGTase. The Asn28 residue at calcium binding site II significantly affects the CGTase specific activities, *k*_cat_ and *k*_cat_/*K*_m_, yet not the thermostability. Residue Asn132 at the calcium binding site I was sensitive to modification, and substitution of it to Arg completely disrupted the CGTase activity. Ionic interactions were introduced in the calcium binding site to replace the function of the calcium ion and stabilize CGTase; however, this was not accomplished. Residue 182, which is adjacent to calcium binding site I, was found to be important for thermostability. Two separate mutations (S182G and S182E) at this position increased CGTase stability. The replacement of Ser182 to Gly and Glu resulted in approximately a 327% and 41% increase in activity half-life at 60 °C, respectively, with the half-life of parent CGTase being only 22 min. In summary, the CGTase activities and thermostability are sensitive to the calcium binding site residues.

## Figures and Tables

**Table 1:** Comparison of optimal temperature and thermostability of various CGTases.

CGTase	Optimum temperature (°C)	Half-life, t_1/2_ (min)	Reference
Parent CGTase (this study)	60	22 min at 60 °C	
*Bacillus circulans* 251	60	9.7 min at 60 °C	[11]
*Bacillus* sp. strain G-825-6	50–55	nd *	[20]
*Bacillus stearothermophilus* ET1	80	10 min at 80 °C	[21]
*Thermoanaerobacterium thermosulfurigenes* EM1	80–95	30 min at 100 °C	[7]
*Thermoanaerobacter* sp. ATCC 53627	95	nd *	[22]

* not determined.

**Table 2:** The specific activities for parent and mutant CGTases.

CGTase	Specific activity (U mg^−1^)		

	β-CD cyclization	γ-CD cyclization	Coupling
Parent	216 (8.11)	1.08 (0.01)	0.67 (0.01)
N28R	359 (23.46)	2.13 (0.04)	0.67 (0.01)
S182G	162 (10.59)	1.08 (0.02)	0.70 (0.03)
S182E	178 (22.29)	0.76 (0.14)	0.77 (0.04)

Each value represents the mean of three independent measurements and the numbers between the brackets indicate the standard deviation.

**Table 3:** The enzyme kinetics for parent and mutant CGTases.

Enzyme	*k*_cat_ (s^−1^)	Efficiency, *k*_cat_/*K*_m_ (mL·mg^−1^·s^−1^)
Parent	3.13 ± 0.13	1.82 ± 0.001
N28R	3.52 ± 0.17	2.52 ± 0.35
S182G	2.62 ± 0.12	2.00 ± 0.05
S182E	2.57 ± 0.22	1.82 ±0.27

**Table 4:** Efficiencies of cyclodextrins formation as determined by HPLC.

Enzyme	Conversion of starch into cyclodextrins (%)				

	Tapioca starch	Soluble starch	Rice starch	Corn starch	Potato starch
Parent	9.01	6.73	7.86	3.31	4.11
N28R	10.26	7.84	9.74	4.78	5.48
S182G	14.01	10.75	13.31	6.48	7.41
S182E	12.01	9.62	10.81	5.49	6.73

Approximately 3 U of CGTases were incubated with 1% of starch for 18 h at pH 6.0 and 60 °C.

**Table 5:** The oligonucleotide primers used in the PCR experiments for the construction of mutagenic CGTase DNA. The primers of EFP_*Bam*HI and ERP_*Hin*dIII are forward and reverse external primers, respectively, and they contain restriction sequences for cloning purposes. The rest are the internal mutagenic primers containing the desired mismatched sequence.

Name of primer	Sequence *
EFP_*Bam*HI	5′-CTCGGATCCGGACGTAACAAACAAAGTCAATTACTC-3′
ERP_*Hin*dIII	5′-GCCAAGCTTCCAATTAATCATAACCGTATCTGTTCCGG-3′
IFP_N28R	5′-CTGGC**cg**CAATCCTTCAGGCGCTATCTTTAG-3′
IRP_N28R	5′-AAGGATTG**cg**GCCAG GATTCCCGTCAGAGAATCG-3′
IFP_N132R	5′-CACGCCA**cgc**CATTCATCACCGGCACTTGAAACG-3′
IRP_N132R	5′-GATGAATG**gcg**TGGCGTGAAATCCATGATTACC-3′
IFP_S182G	5′-GAT**g**GCATTTACAGAAACTTATATGATCTGGCAG-3′
IRP_S182G	5′-GTTTCTGTAAATGC**c**ATCTTCATATGAAGAGAAATCTG-3′
IFP_S182E	5′-GAAGAT**gaa**ATTTACAGAAACTTATATGATCTGGCAG-3′
IRP_S182E	5′-TCTGTAAAT**ttc**ATCTTCATATGAAGAGAAATCTG-3′

* The restriction sequences are underlined and the mismatches are indicated in the bold lower-case letters.
